# Three-dimensional ultrastructure of giant mitochondria in human non-alcoholic fatty liver disease

**DOI:** 10.1038/s41598-021-82884-z

**Published:** 2021-02-08

**Authors:** Gerald J. Shami, Delfine Cheng, Pauline Verhaegh, Ger Koek, Eddie Wisse, Filip Braet

**Affiliations:** 1 School of Medical Sciences (Discipline of Anatomy and Histology), The University of Sydney, Camperdown, NSW 2006 Australia; 2 Maastricht MultiModal Molecular Imaging Institute, Division of Nanoscopy, Maastricht University, Maastricht, The Netherlands; 3 Department of Internal Medicine Division of Gastroenterology and Hepatology, Maastricht University Medical Centre, Maastricht, The Netherlands; 4 Sydney Microscopy & Microanalysis, The University of Sydney, Camperdown, NSW 2006 Australia; 5 Cellular Imaging Facility, Charles Perkins Centre, The University of Sydney, Camperdown, NSW 2006 Australia

**Keywords:** Gastroenterology, Hepatology, Liver diseases, Non-alcoholic fatty liver disease

## Abstract

Giant mitochondria are peculiarly shaped, extremely large mitochondria in hepatic parenchymal cells, the internal structure of which is characterised by atypically arranged cristae, enlarged matrix granules and crystalline inclusions. The presence of giant mitochondria in human tissue biopsies is often linked with cellular adversity, caused by toxins such as alcohol, xenobiotics, anti-cancer drugs, free-radicals, nutritional deficiencies or as a consequence of high fat Western diets. To date, non-alcoholic fatty liver disease is the most prevalent liver disease in lipid dysmetabolism, in which mitochondrial dysfunction plays a crucial role. It is not well understood whether the morphologic characteristics of giant mitochondria are an adaption or caused by such dysfunction. In the present study, we employ a complementary multimodal imaging approach involving array tomography and transmission electron tomography in order to comparatively analyse the structure and morphometric parameters of thousands of normal- and giant mitochondria in four patients diagnosed with non-alcoholic fatty liver disease. In so doing, we reveal functional alterations associated with mitochondrial gigantism and propose a mechanism for their formation based on our ultrastructural findings.

## Introduction

Mitochondria are unique double-membrane bounded organelles that primarily function in cellular respiration via the oxidative phosphorylation of carbohydrates and fatty acids to produce adenosine triphosphate (ATP)^1,2^. The observation of these ubiquitous organelles was first identified by Altmann^3^ by means of light microscopy. However it wasn’t until the early 1950s that the internal ultrastructure of mitochondria was more wholly illustrated by Palade^4^ and Sjöstrand^5^, utilising ultrathin sectioning and electron microscopy.

Mitochondria are structurally characterised by a spherical or elongated ovoid shape, which results in highly variable images depending upon the orientation and plane of sectioning^4^. They are delimited from the cytosol by a smooth *outer membrane* (~ 7 nm thick), and a second *inner membrane* (~ 5 nm thick) to which parallel infoldings of cristae (more fully, *cristae mitochondriales*) are attached via tubes called *pediculi cristae* of varying length^6^. The infoldings of the cristal membrane is functionally important for increasing the surface area for ATP production^7,8^. The concentric arrangement of the inner and outer mitochondrial membranes forms two sub-compartments: the *intermembranous space*^9^, located between the inner and outer membranes; and the *matrix*, which is the space inside the inner membrane. The matrix contains mitochondrial DNA (mtDNA), soluble enzymes of the citric acid cycle, matrix granules, ribosomes, nucleotide cofactors and inorganic ions^9–11^. Nearly all living eukaryotic cells contain mitochondria, however their size (~ 0.5 to 3 µm), shape and number varies considerably, both between different cell types but also within the chondriome (the total mitochondria population of a cell)^12,13^. By observing mitochondria in living cells, it became apparent that they constantly move, change shape and sometimes fuse or split into two daughter mitochondria^14^. Mitochondrial dynamics are essential for the maintenance of their shape, distribution and size, alterations of which—typically associated with mutations in GTPases belonging to the Dynamin family—are associated with numerous human diseases^15^.

Given the cardinal role that mitochondria play in normal cellular function, mitochondrial abnormalities such as gigantism, in which mitochondria can reach diameters greater than that of the cell nucleus, represent an area of particular interest, due to the link between mitochondrial ultrastructural alterations, dysfunction and the development of clinical disease^2,16,17^. Giant mitochondria (GM) (a.k.a. megamitochondria) were first observed in 1958 by Ekholm and Edlund ^18^ in human patients diagnosed with cholestatic liver disease. Not long thereafter, Novikoff and Essner^19^ observed similar structures, describing “electron-opaque deposits in membrane-bound bodies” in rat hepatic parenchymal cells (HPC) in animals injected with bilirubin. At the time, they hypothesised that those “bodies” were mitochondria and might arise as a non-specific response to noxious agents. Throughout the ensuing years, GM have been reported to occur in a variety of hepatopathologies, including: hepatic porphyria^20^, Gilbert’s syndrome^21^, viral hepatitis^22^ alcoholic^23–25^ and non-alcoholic liver disease^26^ to name a few. GM have also been experimentally induced in various animal models proceeding nutritional or pharmacological manipulation^24,27^.

The occurrence of GM is not restricted to the liver, having been reported amongst a variety of tissues—particularly those that display a high degree of metabolic activity, such as cardiac, skeletal, adipose^28^ and neural tissue^29^. GM have also been observed to occur in a variety of species including: mice, rats, gerbils, dogs and monkeys^30–32^. A few studies have reported on GM in normal cells^33^ and apparently normal liver^34^, however the lack of available literature suggests this isn’t a particularly common occurrence.

GM display a highly variable appearance that is commensurate with the diverse milieu amongst which they exist. Their gross morphology can be classified as elongated, irregular or spheroidal^23,35,36^ the frequency of which is correlated with specific diseases and experimental models. The internal configuration of GM is both multifold and dramatically distinct from their normal-sized counterparts. They are typically characterised by a greatly augmented matrix^37^, atypically arranged and/or sparse cristae^38^, dense granules^39,40^, vacuoles^41^, myelin figures^42^ and intramitochondrial inclusions, referred to as crystalline^43^, crystalloid^44^, paracrystalline^45^ or filamentous^46^.

Whilst GM have been characterised in various tissues and disease states, previous studies have primarily utilised conventional two-dimensional (2-D) imaging approaches, providing a limited insight into the complex three-dimensional (3-D) morphology, internal ultrastructure, distribution and relationship of GM with functionally related organelles. Herein, we employ a complementary imaging approach involving array tomography (AT) and transmission electron tomography (TET) in order to comparatively analyse the structure and morphometric parameters of normal mitochondria (NM) and GM in four patients diagnosed with non-alcoholic fatty liver disease. In so doing, we reveal functional alterations associated with mitochondrial gigantism in human hepatic tissue and propose a mechanism for their formation based on 3-D models and ultrastructural findings, in concert with the classification and quantification of GM morphology.

## Results

Herein, we utilised an established methodological approach for preserving human liver biopsies through injection-fixation that permits the full disclosure of fine structure under the most ideal preservation conditions, thereby disclosing meaningful observations at the nanometre scale^47^.

**Figure 1:**
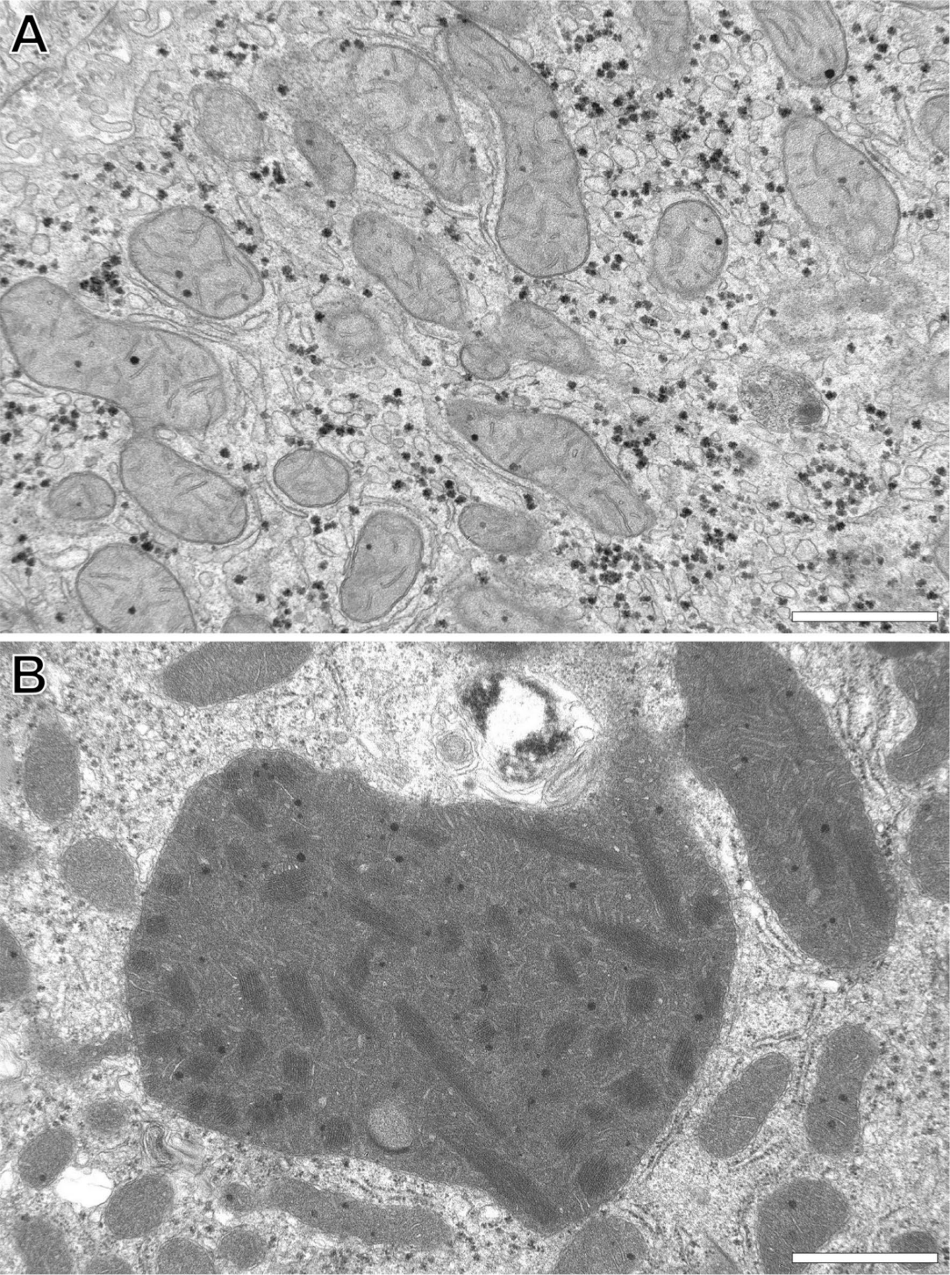
Representative transmission electron microscopy images of human liver parenchymal mitochondria. (**A**) Reveals normal-shaped and -size mitochondria versus (**B**) which shows a giant mitochondrion that is many times larger relative to normal mitochondria and displays a distinctly altered internal fine structural appearance. Images were obtained from ultrathin sections of injection-fixed wedge biopsies and captured in two-dimensions at intermediate magnification (19,000×) through the use of conventional transmission electron microscopy. Scale bar = 1 µm.

Tissue sections were consistently examined first at low magnifications (~ 50× – 120×) prior to the use of higher magnifications (up to 90,000×) using transmission electron microscopy, allowing the rigorous observation of a large number of HPCs (*n* =  ~ 200 to 300) in a single field-of-view. This 2-D imaging approach facilitated the detailed investigation of HPCs within the different biopsies containing giant mitochondria. In total, 5014 TEM images were recorded from 57 patients, of which 32 contained GM. In those sections, GM appear in different shapes and sizes when compared to NM (compare Fig. 1A vs. B). GM can be almost cylindrical and elongate, sometimes irregularly branched or spheroidal. Moreover, the matrix content of GM presents as significantly distinct from NM observed in static EM images.

In order to study the peculiar organisation of those giant mitochondria, we next randomly selected 4 biopsies as representative examples for further detailed structural analysis in 3-D at the nanometre resolution. 3-D EM analysis as employed herein has the obvious advantage of collecting unseen detail at high lateral and axial resolution relative to other imaging approaches. The observations detailed below represent approximately 288 h of beam-time and over 800 h of subsequent data modelling and analysis.

### Classification of giant mitochondria

We first aimed to classify GM in HPCs on the basis of morphological deviations relative to NM ultrastructure. Specifically, GM were designated as those containing sparse or irregularly arranged cristae, enlarged matrix granules and filamentous intramitochondrial crystalline inclusions (ICIs) (Fig. 1B). Of note, GM were not observed in non-parenchymal cells of the liver, such as liver sinusoidal endothelial cells, Kupffer cells and hepatic stellate cells.

### Comparative morphometry of normal versus giant mitochondria

**Figure 2:**
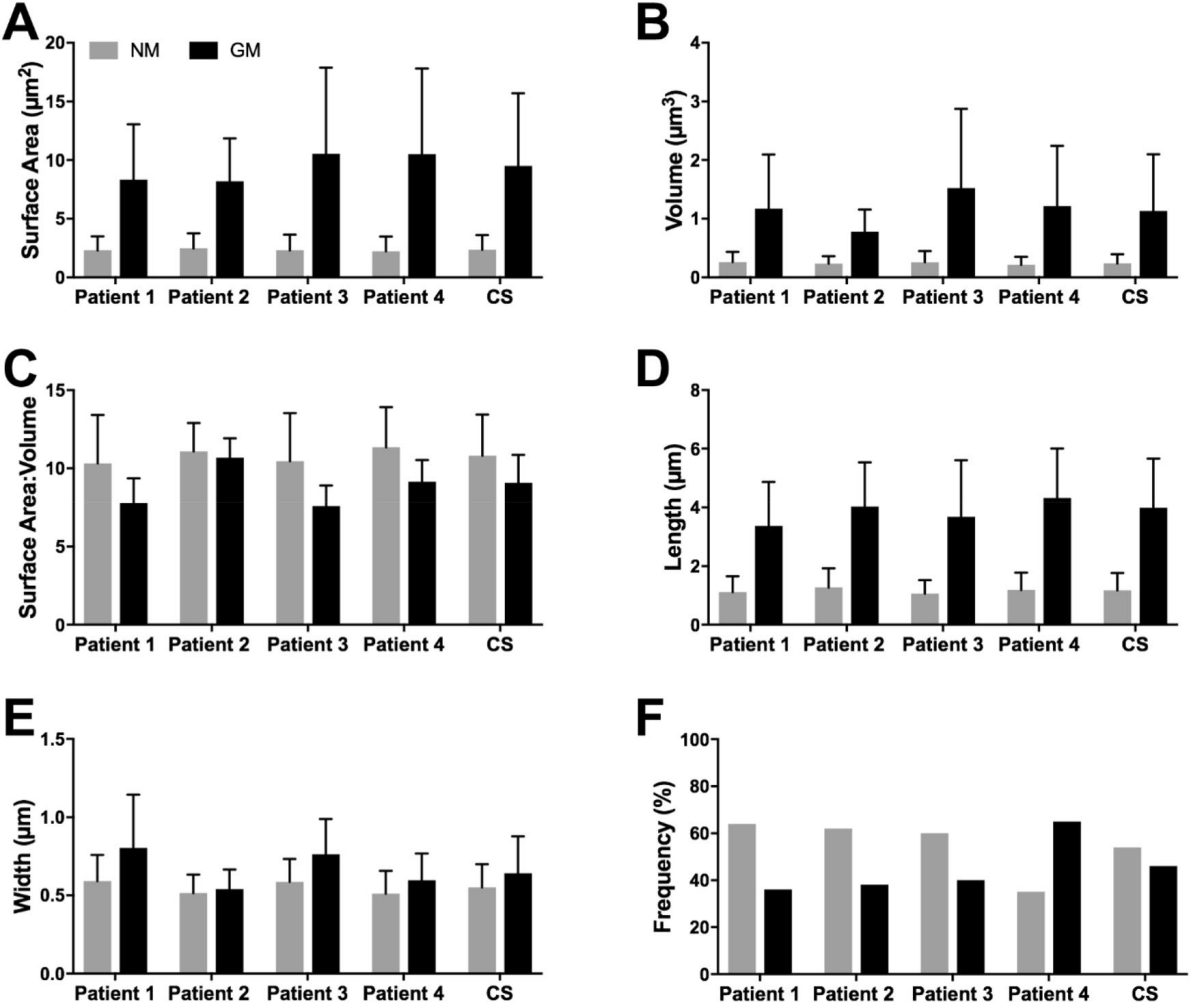
Quantitative comparison of a range of common morphometric parameters obtained from normal- (*n* = 2451) and giant mitochondria (*n* = 2081). (**A**) Mean giant mitochondria surface area (9.51 ± 6.20 µm^2^) was 4 × greater relative to normal mitochondria (2.35 ± 1.26 µm^2^). (**B**) On average giant mitochondria volume (1.13 ± 0.97 µm^3^) was 4.7 × larger than normal mitochondria (0.24 ± 0.15 µm^3^). (**C**) Surface area-to-volume ratio was 17.4% reduced between giant mitochondria and normal mitochondria. (**D**) Mean giant mitochondria length measured 3.99 ± 1.68 µm versus 1.18 ± 0.59 µm in normal mitochondria. (**E**) On average giant mitochondria were ~ 20% wider than normal mitochondria (0.64 ± 0.24 µm vs. 0.55 ± 0.15 µm). (**F**) The frequency of giant mitochondria versus normal mitochondria ranged between 36 and 65%, with a combined average of 46% for all patients. For each parameter statistical significance using Student’s t-test, *P* =  ≤ 0.001. One-way ANOVA indicates a lack of inter-patient variability for the NM groups, *P* =  < 0.0001. Additional tabular and graphical data derived from each of the 16 cells analysed (4 cells/patient) is also available (Supplementary Data S2–S4). Legend: CS, Combined score of all patients.

Figure 2 graphically summarises a variety of common morphometric parameters derived from all patients, for NM (green) (54%; *n* = 2451) and GM (red) (46%; *n* = 2081) illustrated in Supplementary Figure 1. The data reported below are the total means ± S.D, combining all respective measurements from each of the patients. All comparative measurements were considered statistically significant (*P* =  < 0.001). Additional tabular and graphical mitochondrial data derived from each of the 16 cells analysed is also available (Supplementary Data S2–S4).

Mean GM surface area (9.51 ± 6.20 µm^2^) and volume (1.13 ± 0.97 µm^3^) measurements were 4 × and 4.7 × greater, respectively, relative to NM (µ_x_ NM surface area = 2.35 ± 1.26; µ_x_ NM volume = 0.24 ± 0.15 µm^3^) (Fig. 2A,B). Of note, this represented a 17.4% reduction in the surface area-to-volume (SA:V) ratio between GM and NM (Fig. 2C). In rare instances (< 1%), GM were observed to reach incredible volumes of up to 26% of the mean HPC nucleus volume (277.92 ± 139.23 µm^3^; *n* = 19) and 1.15% of the average HPC volume (6443.81 ± 5037.50 µm^3^; *n* = 16).

On average GM were 3.5 × longer (µ_x_ GM length = 3.99 ± 1.68 µm; µ_x_ NM length = 1.18 ± 0.59 µm) and 1.2 × wider than NM (µ_x_ GM width = 0.64 ± 0.24 µm; µ_x_ NM width = 0.55 ± 0.15 µm) (Fig. 2D,E). Mitochondrial length was defined as the straightest path along the length of a given mitochondrion, according to the criteria established by Noske, et al. ^48^. No attempts were made to distinguish between NM or GM in a state of fusion or fission; thus, mitochondrial length is reflective of calliper measurements (i.e. diameter max), and not total mitochondrial length, incorporating branching/fusing segments. GM were rarely observed to measure tremendous lengths of up to 13.36 µm, representing 44% of the average HPC length (30.41 ± 9.15 µm; *n* = 16).

Mean patient frequency measurements of GM, relative to NM, ranged between 36 and 65%, with a combined average of 46% for all patients (Fig. 2F, Supplementary Data S4). The highest GM frequency was observed in a cell from patient 4, of which 76% of the chondriome was composed of GM.

### Whole cell reconstruction of the entire chondriome

**Figure 3:**
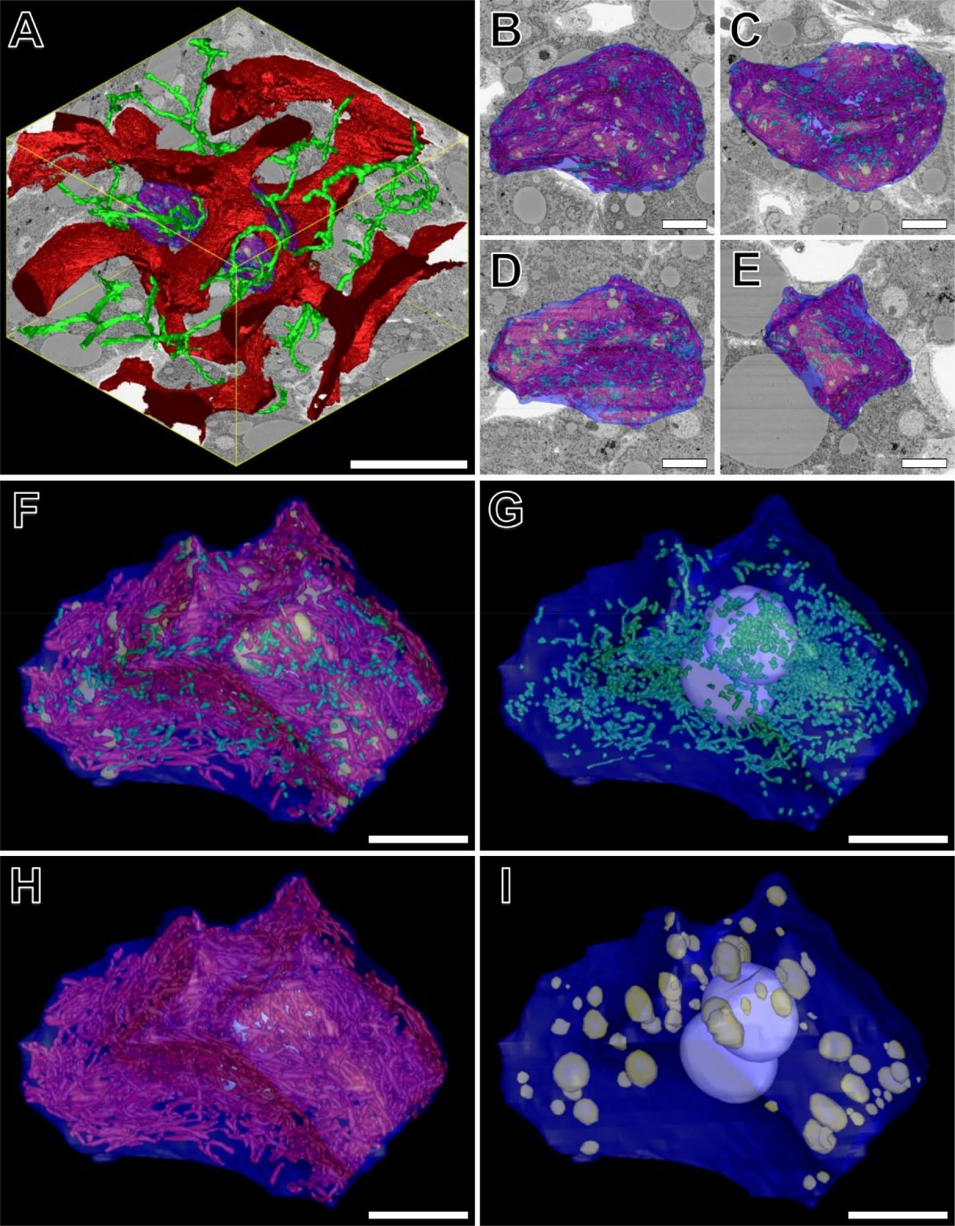
(**A**) Reconstructed 3-D volume consisting of 400 consecutive images (section thickness = 150 nm). *XY* = 84.82 µm, *Z* = 60 µm. Total volume = 453,249 µm^3^. Magnification 1200×. The volume contains a 3-D reconstruction of a hepatic parenchymal cell (HPC) (blue) from patient 4, surrounded by the hepatic sinusoidal network (red) and bile canaliculi (green). (**B** and **C**) show top and bottom views, respectively, of the HPC of interest, overlayed with an *XY* inverted backscattered SEM (BSEM) image. (**D** and **E**) reveal front and right-side model views, respectively, overlayed with corresponding *XZ* and *YZ* BSEM images. (**F**–**I**) reveals higher-magnification model views of the HPC and corresponding model views of normal- (green) and giant mitochondria (red), lipid droplets (yellow) and nuclei (white). Scale bars: (**A**) = 30 µm; (**B**–**I**) = 10 µm.

Segmentation, modelling and morphometric analysis of the entire chondriome—selected from a cell of interest derived from patient 4—was performed in order to comprehensively visualise (Fig. 3 and Supplementary Video 1) and quantify (Table 1) NM and GM, with functionally related structures of interest. The cellular organisation of both NM and GM were evenly distributed throughout the cytoplasm. GM displayed three directions of polarity (Fig. 3H), which were aligned with the three longitudinal facets of the sinusoid-HPC plasma membrane domain (Fig. 3A). Whilst normal-sized mitochondria revealed a similar directionality, this was less evident due to their smaller size (Fig. 3G).

**Table 1:** A comparative summary of quantitative volumetric data, extending the image data for the selected hepatic parenchymal cell depicted under Fig. 3.

	HPC	Mitochondria		Lipid droplets	Nuclei
		Normal	Giant		
Total number	1	1142 (45%)	1384 (55%)	106	2
Total SA	4107.73 µm^2^	2461.50 µm^2^	12,797.52 µm^2^	1223.27 µm^2^	621.60 µm^2^
Mean SA	N/A	2.15 ± 1.31 µm^2^	9.25 ± 7.00 µm^2^	11.54 ± 13.97 µm^2^	310.80 ± 9.26 µm^2^
Total volume	15,584 µm^3^	241.24 µm^3^	1511.08 µm^3^	533.98 µm^3^	1011.22 µm^3^
Mean volume	N/A	0.21 ± 0.14 µm^3^	1.09 ± 1.51 µm^3^	5.04 ± 9.11 µm^3^	505.61 ± 22.26 µm^3^
% of HPC volume	N/A	1.55%	9.70%	3.43%	6.49%

HPC, Hepatic parenchymal cell; SA, surface area. Results are expressed as means ± S.D where applicable.

Both NM and GM were frequently observed in close association with lipid droplets and glycogen rosettes and were regularly seen to surround larger lipid spheres. The outer mitochondrial membrane was frequently in such close association with the outer lipid surface that the two structures appeared to fuse. These observations are commensurate with their prime function in the production of ATP, of which both fatty acids—derived from lipid droplets—and carbohydrates—derived from glycogen—are primary substrates. Another common association observed was between mitochondria and the rough endoplasmic reticulum (rER), of which curved profiles of the latter were seen to partially or fully surround mitochondria. Broad areas of direct contact between the two structures was a frequent observation.

A summary of quantitative data calculated from the 3-D models of mitochondria, lipid droplets and HPC nuclei within the selected cell of interest has been summarised in Table 1. The binucleate HPC appeared hypertrophic, being 3.8 × more voluminous (HPC volume = 15,584 µm^3^) than the median value (4493.20 µm^3^; *n* = 16). The two HPC nuclei had a volume of 521.35 µm^3^ and 489.87 µm^3^ (µ_x_ nucleus volume = 505.61 ± 22.26 µm^3^) and occupied 6.49% of the cytoplasmic volume.

The chondriome of the cell of interest contained a total of 2526 mitochondria, of which 45% (*n* = 1142) were normal-sized, and 55% (*n* = 1384) were giant. The total chondriome occupied 11.25% (1752.32 µm^3^) of the cytoplasmic volume. NM only occupied 1.55% of the HPC cytoplasmic volume (total NM volume = 241.24 µm^3^), whilst GM constituted the remaining 9.70% (total GM volume = 1511.08 µm^3^). Mean GM surface area (9.25 ± 7.00 µm^2^) and volume (1.09 ± 1.51 µm^3^) measurements were 4.3 × and 5.2 × greater, respectively, relative to NM (µ_x_ NM surface area = 2.15 ± 1.31 µm^2^; µ_x_ NM volume = 0.21 ± 0.14 µm^3^) (Table 1). These measurements did not statistically differ from the mitochondrial measurements reported for the sampled cells—derived from each patient—thus validating the sampling protocol employed in this study.

The HPC of interest contained 106 lipid droplets that were peripherally distributed throughout the cytoplasm, in close proximity to the HPC plasma membrane. Lipid droplets varied considerably both in surface area (µ_x_ 11.54 ± 13.97 µm^2^; total = 1223.27 µm^2^) and volume (µ_x_ 5.04 ± 9.11 µm^3^; total = 533.98 µm^3^), occupying 3.43% of the HPC cytoplasmic volume.

### Characterisation of giant mitochondria morphology

**Figure 4:**
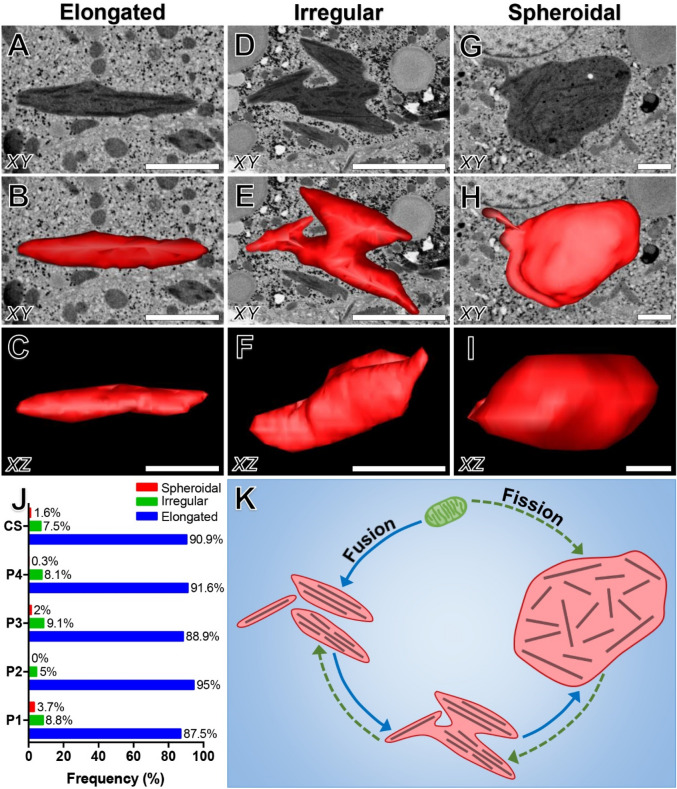
A comparative overview of the three most common giant mitochondria morphologies, and proposed mechanism for their formation. (**A–C**) Elongated mitochondria revealing a spindle or rod-shaped morphology. They are characterised by ICIs arranged parallel relative to the longitudinal axis of the mitochondrion. (**D**–**F**) Irregular mitochondria displaying a branching or stellate morphology in which ICIs are oriented parallel to the longitudinal axis of the various branching/fusing segments. (**G**–**I**) An enormous spheroidal giant mitochondrion displaying a globose morphology in which numerous mitochondrial inclusions are visible throughout the matrix, and ICIs are oriented with no particular directionality. (**J**) Quantitative comparison of the relative frequency of elongated, irregular and spheroidal giant mitochondria amongst the four patients. Legend: P1-4, Patient 1–4; CS, indicates the combined score for all patients. (**K**) Proposed mechanism for the formation of giant mitochondria. Giant mitochondria may form by either fusion (blue arrows) of elongated mitochondria or fission of spheroidal mitochondria (green dotted arrows), or both. Fusion involves initial degeneration of normal mitochondria, accompanied by disorganisation of cristae and the parallel alignment of ICIs relative to the longitudinal axis, to form elongated mitochondria. Next, multiple elongated mitochondria aggregate and fuse to form irregular mitochondria, in which ICIs are arranged parallel relative to the longitudinal axis of each branching/fusing segment. Irregular mitochondria appear to accumulate additional degenerative alterations, and in so doing enlarge to form spheroidal mitochondria, which are characterised by paucity of the cristal membrane and a random arrangement of ICIs throughout the mitochondrial matrix. The proposed mechanism for giant mitochondria formation via fission (green dotted arrows) involves gross enlargement of normal mitochondria into spheroidal mitochondria. These may rearrange into branching profiles (irregular mitochondria), which then may divide into multiple elongated mitochondria. Based on ultrastructural observations and frequency measurements, the mechanism for mitochondrial fission as the primary mechanism for giant mitochondria formation seems unlikely, however cannot be excluded. Scale bar: 3 µm.

GM were categorised into three general types based on the previous descriptions of Iseri and Gottlieb ^35^. Figure 4 illustrates the various 3-D morphologies observed and quantifies their relative frequencies (*n* = 2081) amongst the patients analysed (Fig. 4J).

*Elongated giant mitochondria* (Fig. 4A–C and Supplementary Video 2) were spindle- or rod-shaped as was consistent with the observations of Uchida et al.^23^, and were the most frequently occurring morphology (90.9%) (*n* = 1892) (Fig. 4J). On average they measured 3.90 ± 1.63 µm in length—however were seldom observed to reach lengths of up to 12 µm (< 1%)—0.63 ± 0.20 µm in width and 1.02 ± 0.79 µm^3^ in volume. Organisation of the cristae was highly variable, ranging from well-developed to completely disorganised amongst all patients. ICIs were characteristically oriented parallel to the longitudinal axis of elongated mitochondria, and when sectioned in perfect longitudinal section, could be visualised extending the full length of the mitochondrion.

*Irregular giant mitochondria* were characterised by a branching or stellate morphology (Fig. 4D–F and Supplementary Video 3) and were of moderate frequency (7.5%) (*n* = 156). On average they measured 4.89 ± 2.07 µm in length, 0.71 ± 0.46 µm in width and 2.00 ± 1.42 µm^3^ in volume. Irregular mitochondria typically revealed increased cristal membrane disorganisation relative to elongated mitochondria. Organisation of ICIs were observed in various orientations, running parallel to the longitudinal axis of branching segments (Fig. 4D).

*Spheroidal giant mitochondria* were circular or globular in morphology (Fig. 4G–I and Supplementary Video 4) and the least frequently occurring morphology (1.6%) (*n* = 33) (Fig. 4J). They were observed in all patients except patient 2. They were particularly conspicuous, frequently rivalling the dimensions of the nucleus (Fig. 4G). They measured 6.64 ± 2.05 µm in length, 2.40 ± 1.68 µm in width and 20.23 ± 19.21 µm^3^ in volume. Of the three morphologies characterised, spheroidal mitochondria displayed the most bizarre internal configuration. Cristal membrane disorganisation was particularly pronounced, and numerous atypical inclusions were observed within the mitochondrial matrix, including: electron-lucent vacuoles, enlarged electron-dense matrix granules, lipidic material and ICIs randomly oriented with no specific directionality, in opposition to the regular arrangement of such structures in elongated and irregular mitochondria.

### Electron tomographic characterisation of intramitochondrial crystalline inclusions (ICIs) and enlarged matrix granules

**Figure 5:**
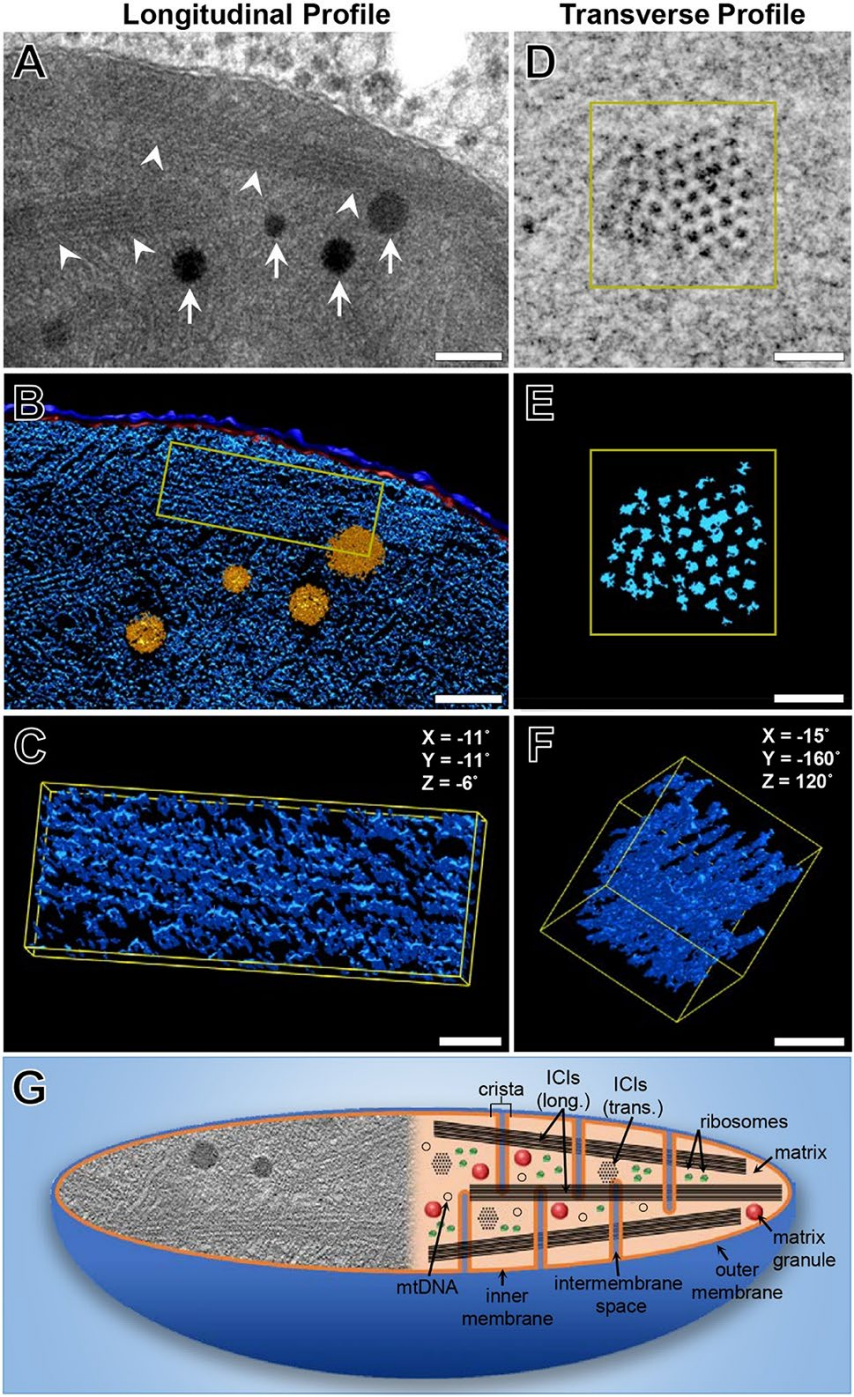
The 3-D structure of intramitochondrial crystalline inclusions (ICIs) and enlarged matrix granules revealed by transmission electron tomography. (**A**) High-magnification image (15,000× magnification) of longitudinal arrays of ICIs (arrowheads) and enlarged matrix granules (arrows) distributed throughout the mitochondrial matrix. (**B**) 3-D model view of the outer- (dark blue) and inner mitochondrial membranes (red), ICIs (light blue) and enlarged matrix granules (orange), corresponding to (**A**). (**C**) Higher-magnification model view of a bundle of ICIs corresponding to the yellow bounding box in (**B**). (**D**) High-magnification image (30,000× magnification) and corresponding model view (**E**) of a bundle of ICIs in transverse section. The individual rod-like filaments appear to be composed of globular subunits, measuring 8.65 ± 1.31 nm in diameter and are arranged in a rhomboid pattern with regular periodicities of 7.38 ± 1.61 nm. (**F**) Rotated model view relative to (**E**) indicating the rod-like inclusions are organised as individual filaments, and not lamellar sheets as may be perceived from 2-D TEM micrographs. (**G**) Schematic diagram revealing the internal ultrastructure of giant mitochondria. The left-hand side of the diagram reveals a 2-D image of common ultrastructural abnormalities within the matrix of giant mitochondria, such as ICIs enlarged matrix granules and disrupted cristae. The right-hand side of the diagram reveals characteristic features of giant mitochondria that may not be observed in their entirety in a single image, or a single magnification, including: mitochondrial DNA, ribosomes, matrix granules, the inner- and outer mitochondrial membranes and intervening intermembrane space, as well as ICIs in both longitudinal and transverse profile. Scale bars: (**A** & **B**) 200 nm; (**C**–**F**) = 50 nm.

ICIs were a prominent feature characteristic of HPC mitochondria, whose appearance was highly dependent on the plane of sectioning with respect to the plane of the crystal lattice. In longitudinal section, ICIs appeared as parallel arrangements of filamentous or rod-like inclusions (Fig. 5A). ICIs were organised into bundles that, if captured in the appropriate orientation, could be visualised spanning the full length of the mitochondrion. The direction and organisation of ICIs within the mitochondrial matrix was highly dependent on the specific gross morphological classification of an individual mitochondrion (elongated, irregular or spheroidal) as outlined above.

In cross-section, ICIs appeared as highly ordered arrays of globular, dot-like structures of homogenous electron-density. Individual filaments measured 8.65 ± 1.31 nm (*n* = 100) in diameter and were arranged in an equilateral rhomboid pattern with regular periodicities of 7.38 ± 1.61 nm (*n* = 100) (Fig. 5D,E). The intervening space between filaments was of similar electron-density to the surrounding mitochondrial matrix. Transversely, conglomerates of ICIs were arranged as cylindrically shaped bundles measuring 140.95 ± 16.46 nm (*n* = 50) in diameter (Fig. 5D–F). The number of filaments per bundle was highly variable. Bundles of ICIs were located within the mitochondrial matrix, and often observed in close association with cristae, including the intercristal space. ICIs were not observed within the intracristal or intermembranous compartments.

Another prominent feature of GM was the presence of enlarged matrix granules, which were particularly prominent within “irregular” and “spheroidal” GM. These appeared as large electron-opaque spherules, composed of smaller amorphous subunits. Enlarged matrix granules measured 117.56 ± 25.65 nm in diameter, representing a 2.8 × increase relative to matrix granules derived from NM (µ_x_ NM granule diameter = 42.59 ± 8.57 µm) (Fig. 5A,B). No visible differences in the electron density of both normal-sized and enlarged matrix granules were observed.

## Discussion

Mitochondrial function is intrinsically tied to normal ultrastructure. In many liver diseases mitochondrial structural abnormalities are often attributed to alterations in metabolism and an increase in oxidative stress. The common observation of GM in non-alcoholic fatty liver disease^49^ is attributed to cellular lipid accumulation and increased β oxidation in the formation and breakdown of triglycerides. In the present study, we aimed to investigate structural mitochondrial alterations in patients diagnosed with NAFLD.

We first aimed to classify GM which has been a topic of debate since their original description in 1964 and remains largely unanswered to-date. Structural parameters such as size and fragmented appearance for example have been considered but have proven inadequate as gigantism is correlated with ultrastructural alterations relative to normal mitochondria. Fragmented mitochondria on the other hand are considered normal if they do not reveal ultrastructural features associated with giant mitochondria (i.e. intracrystalline filaments, enlarged electron-dense granules etc.). Noteworthy, fragmentation of normal-appearing mitochondria is likely to indicate a state of fusion or fission, in which their size would be larger than usual. Hence, size or fragmented appearance were not used as indicators to designate mitochondria as “normal” or “giant” in this study.

Array tomography expedited the acquisition of large cellular volumes at the nanometre scale, facilitating the global reconstruction of thousands of NM and GM and accompanying morphometric data. Collectively, the relative quantitative differences between NM and GM revealed significant differences amongst the most commonly used morphometric parameters outlined under Fig. 2 and Table 1. Of particular note was the 17.4% reduction in the SA:V ratio of the outer mitochondrial membrane between GM and NM; suggesting a potential reduction in the efficiency and utilisation of pyruvate (the product of glycolysis) and free fatty acids within the cytosol, through the outer mitochondrial membrane. This alteration is particularly profound considering that GM occupied 6.25 × greater cytoplasmic volume of the HPC of interest (Fig. 3), despite only accounting for a 10% increase in number, relative to NM. Impaired energy-producing capacity of GM relative to their normal-sized counterparts, was further supported by our observations of disorganisation and paucity of the inner mitochondrial membrane; the infoldings of which—into cristae—are well-documented to increase the surface area for ATP production^50^. Furthermore, abnormal intramitochondrial inclusions such as ICIs and enlarged matrix granules, were observed to occupy a significant portion of the matrix of GM (~ 30 to 40%), impeding upon the space available for the various reactions of the citric acid cycle to occur.

Despite gross morphological and ultrastructural alterations indicating deranged mitochondrial metabolism, GM function does not appear to be completely lost. Reconstruction of the entire chondriome of a randomly selected cell of interest (Fig. 3) revealed parallel alignment of GM within the cytoplasm, along the three longitudinal axes of the sinusoid-HPC plasma membrane domain (Fig. 4A). These observations are commensurate with the suggestion that mitochondrial orientation is dependent upon the direction of diffusion currents within cells^51^. Functionally, the arrangement of GM in such a manner, is important in reducing the distance for diffusion- and increasing surface area for the uptake of oxygen and primary substrates for ATP production, from their source in the hepatic sinusoids, to their site of utilisation in mitochondria^52^. Retention of some degree of mitochondrial function is further supported by the close association between GM and lipid droplets, glycogen rosettes and the rER. Lipid droplets are a well-documented source for the maintenance of mitochondrial membrane integrity and fatty acids for mitochondrial β oxidation, and have more recently been shown to play a protective role in the sequestration of toxic lipids that arise during autophagic degradation of membranous organelles^53^. Similarly, close proximity between glycogen and mitochondria reduces the distance for the uptake of pyruvate at the outer mitochondrial membrane, from glycogen depolymerisation into glucose and conversion by means of glycolysis within the cytosol. Mitochondrial association with the rER is another common phenomenon implicated in a variety of biological processes, including: mitochondrial fusion, Ca^2+^ transfer, autophagy and inflammasome formation^54^.

After rigorous analysis of thousands of normal- and giant mitochondria, a continuity between the three GM morphologies (elongated, irregular and spheroidal) appears to exist, from which two mechanisms of GM formation can be postulated. GM may form either by (1) fusion of multiple mitochondria, or (2) by enlargement of a single mitochondrion, or both^34^ (Fig. 4K); of which our data supports the former argument. Initially, the internal ultrastructure of normal-sized mitochondria appears to disorganise, in which mitochondrial membrane irregularities and the accumulation of ICIs parallel to the longitudinal axis of the mitochondrion are observed, to form elongated GM (Fig. 4A). Next, migration and contact of multiple elongated mitochondria is observed in which groups of elongated mitochondria become interlocked forming bizarrely shaped, irregular mitochondria in which multiple fusing profiles are observed (Fig. 4D). Such observations of mitochondrial fusion events are consistent with studies employing live cell imaging to reveal mitochondrial dynamics^55^. ICIs are aligned parallel to the longitudinal axis of the fusing segments, and further degeneration of the cristae membranes is observed, relative to elongated GM. The rare occurrence of enormous spheroidal GM appears to be an indicator of advancing disease progression (Fig. 4G). These structures are characterised by numerous abnormal intramitochondrial inclusions, and most notably contain ICIs which are orientated in various directions throughout the mitochondrial matrix. Whilst the direction of ICIs relative to the longitudinal axis of GM appears to be specific to GM morphology (elongated, irregular or spheroidal), is it unclear whether this is symptomatic or causative of morphology. Projections of regularly arranged cristae throughout the entirety of the mitochondrial matrix are also completely lost. The gross enlargement and accumulation of atypical ultrastructural features indicates further disorganisation of these structures relative to elongated and irregular GM.

The sequence of GM formation via fusion from elongated to irregular and bizarre in morphology is further supported by the observation of intermediate forms, and quantitatively justified by the descending order of frequency for each morphology. Whilst not totally excludable, the reverse mechanism of GM formation via fission, from grossly enlarged spheroidal, to irregular and elongated seems unlikely due to the rare occurrence (≤ 3.7%) of spheroidal GM. If GM formation via fission were to be the dominant mechanism, the division of spheroidal GM into irregular and subsequently elongated mitochondria must be a highly dynamic process.

Throughout the literature, much ambiguity and conflicting descriptions regarding the 3-D nature of intramitochondrial crystals and crystalloids exists. By employing tilt-based TET, we revealed the 3-D structure of ICIs and enlarged matrix granules at a resolution not conferred by AT (Fig. 5). 3-D modelling facilitated the virtual manipulation of ICIs enabling the accurate measurement of the diameter of these structures, and their regularly spaced periodicities. Significant variations throughout the literature regarding the diameter of individual filaments (6–12 nm) and the intervening space (5–20 nm) are likely due to inaccuracies introduced by oblique sectioning and the interpretation of 2-D micrographs^56^. TET revealed ICIs as individual rod-like inclusions composed of globular subunits that were arranged into bundles, the orientation of which was dependent on the classification of GM as previously outlined. Filamentous inclusions were not observed as plate-like or lamellar arrangements, as has previously been documented^57^. Such variations in ICI structure and mitochondrial location may be attributed to specific cell types and disease states and delays in tissue fixation after biopsy or post-mortem^58^. From our observations, ICIs were exclusively limited to the intercristal and matrix compartments, whereas in neuronal and muscular mitochondriopathies they have additionally been observed within the intracristal and intermembranous spaces^58,59^.

The chemical composition and significance of ICIs continues to remain elusive amongst the various pathologies in which GM are observed. Optical diffraction studies in human HPCs have suggested that ICIs represent crystalline phase transitions of the lamellar phospholipid bilayer^60^. The study of Caldwell et al.^26^ was in agreement with these observations, who also demonstrated the presence of ICIs in NAFLD. In a recent study by Nürnberger, et al. ^61^ the authors revealed ICIs of equine chondrocytes were proteinaceous in nature, detecting the protein-relevant elements nitrogen, sulphur and phosphor by means of energy-filtered TEM. It has been postulated that such crystals in hibernating animals and oocytes may represent a form of protein storage, however in human HPCs, the presence and frequency of ICIs is considered an indication of mitochondrial degeneration^34^.

To-date, a major limitation of the studies attempting to characterise the chemical composition of ICIs has been the use of chemically fixed and resin-embedded samples. Future studies should employ cryo-fixation methods such as plunge- or high-pressure freezing in order to determine the unaltered chemical nature of ICIs. Elemental dispersive X-ray diffraction or cryo-atom probe tomography represent two viable techniques for determining the composition of ICIs in vivo at atomic resolution. Despite the advantages cryogenic fixation confers for the near native preservation of cellular ultrastructure, logistical difficulties prohibit its use in clinical settings; hence experimental animal or cell models in which GM are induced represent a viable alternative.

TET revealed a 2.8-fold increase in the diameter of matrix granules present in GM, relative to NM. Previous investigation of mitochondrial granules has revealed that they are composed of calcium, magnesium, phosphorous, phospholipids (cardiolipin), glycolipids, protein and cytochrome c oxidase^62,63^. Large electron-opaque granules measuring similar diameters as reported in this investigation (> 100 nm) were observed by Lehninger, et al. ^64^ in the matrix of rat liver mitochondria incubated in a solution containing calcium and inorganic phosphate. Whilst compositional analysis was not performed on enlarged matrix granules in this study, the increased diameter of these structures may be attributable to alterations in mitochondrial metabolism, resulting in the accumulation of the various afore mentioned components of matrix granules.

## Conclusion

We have complementarily employed array tomography and transmission electron tomography to comparatively analyse normal- and giant mitochondria, in four patients diagnosed with non-alcoholic fatty liver disease. In so doing, we reveal ultrastructural alterations associated with giant mitochondria function, including a significant reduction in the surface area-to-volume ratio and disorganisation of the inner- and cristal mitochondrial membranes indicating impaired function. Whole cell reconstruction of normal- and giant mitochondria, lipid droplets and nuclei provided an in-depth view, illustrating the global distribution and morphometry of such structures in their entirety. By characterising the internal ultrastructure and classifying giant mitochondria morphology (elongated, irregular and spheroidal) our results indicate a continuum in the formation of these anomalous structures via mitochondrial fusion. Finally, transmission electron tomography revealed the three-dimensional structure of intramitochondrial crystalline inclusions, as filamentous rod-like inclusions distributed throughout the mitochondrial matrix and intercristal space. Further studies on the chemical composition of such inclusions is necessary to fully elucidate their biochemical nature and pathophysiological significance in NAFLD and other liver diseases that express GM.

## Materials and methods

### Human NAFLD samples

In this study, fifty-seven wedge biopsies were studied from patients that underwent partial hepatectomy at Maastricht University Medical Centre between September 2005 and September 2009. 32 of the 57 biopsies acquired revealed the presence of GM, of which 4 samples were selected based on a positive double-blind diagnosis for NAFLD made by a clinical pathologist for subsequent 3-D ultrastructural analysis. The study was performed in accordance with the ethical standards of the Declaration of Helsinki, and written informed consent was obtained from each patient. The study was approved by the Medical Ethical Committee of the Maastricht University Medical Centre (Approval number: NCT02422238). Investigation of healthy subjects was out of the scope of this investigation as the collection of tissue biopsies in healthy subjects in not a standard routine practice.

### Sample preparation for electron microscopy

Human liver wedge biopsies measuring ~ 1 cm × 1 cm × 1 cm were fixed with 1.5% glutaraldehyde in 0.067 M sodium cacodylate buffer (pH 7.4) (primary fixative) by means of injection perfusion fixation as previously described in detail^47^. Following the injection of the fixative which induces discolouration and hardening of the soft tissue starting at 20 s, tissues were cut into 1 mm × 1 mm × 1 mm blocks and allowed to react in the primary fixative for no longer than 20 min proceeding injection. Tissues were washed with 0.2 M sodium cacodylate buffer (pH 7.4) and post-fixed in 1% osmium tetroxide in 0.2 M phosphate buffer (pH 7.4) for 1 h at room temperature in darkness. Samples were washed with distilled water and dehydrated in an ascending series of ethanol concentrations starting at 70%. Following complete dehydration, samples were infiltrated with 50% Epon in absolute ethanol overnight, 100% Epon for 1 h and 100% Epon overnight. Tissues were transferred to BEEM capsules in 100% Epon and polymerised at 60 °C for 67 h.

### Array tomography (AT)

In order to reconstruct large cellular volumes, hundreds of serial sections per sample were collected in accordance with the methods developed by Micheva and Smith ^65^. Using a dissector blade, Epon-embedded blocks were trimmed forming a trapezoid approximately 2 × wider than the height of the block. A sparring mixture of Welwood glue and xylene (1:2) was applied to the long edge of the trapezoid and allowed to dry for 2 min. The application of the glue/xylene mixture was essential for adhesion between consecutive sections, ensuring the holistic reconstruction of 3-D structures from a series of 2-D sections^66^.

For each sample, a series of 400 consecutive sections of 150 nm-thick sections (depth sectioned =  ~ 60 µm/sample) was collected on a hydrophilized glass slide by means of glow discharging for 30 s. The slide was then placed on a hot plate (~ 60 °C), allowing the water to evaporate and the sections to stretch out and firmly adhere to the slide (2 min). Next, the glass slide was carbon-coated (15 nm) in order to render it electrically conductive and mounted on a SEM stub. Silver paint was applied from the top surface of the slide, to the underlying stub to further improve conductivity.

Inverted backscattered field emission scanning electron microscopy (BSEM) was conducted using a Zeiss Sigma, operating at 4 kV at a working distance of 5.3 mm. Images (8 K × 8 K pixels, 16-bit, 11.9 × 11.9 × 150 nm voxel dimensions, pixel dwell time 3 µs) were acquired at 1200× magnification yielding a *XY* field of view of 98.4 µm^2^.

In order to achieve anatomically consistent image registration over the 400 images captured for each dataset, a series of digital fiducial markers were manually aligned with cellular features of interest that change predictably between successive sections, such as parenchymal cell nuclei. This facilitated coarse alignment of images, accounting for rotational differences of regions of interest between serial sections, due to imperfectly straight ribbons, section compression and shearing caused by sectioning.

Proceeding data acquisition, AT datasets were processed using Fiji, a freeware open-source image processing software package^67^. Datasets were converted to 8-bit pixel depth and resampled to a final voxel size of 37.5 × 37.5 × 150 nm by means of average pixel binning. Image histogram stack normalisation was performed, in order to improve global image contrast. Images were automatically aligned using the StackReg plugin for Fiji^68^ and finally cropped to produce a symmetrical 3-D dataset.

### Transmission electron tomography (TET)

In order to reconstruct the fine internal ultrastructure of GM, 120 nm-thick sections were generated from the same blocks previously sectioned and mounted onto 200 mesh copper grids. Sections were then post-stained with 2% aqueous uranyl acetate and Reynold’s lead citrate for 10 min each.

Two tomograms were acquired at 15,000× and 30,000× magnifications respectively, using a JEM-2100 (Jeol, Japan) transmission electron microscope operating at 200 kV. Single axis tilt series were captured with a bottom-mounted UltraScan 4000 large-format CCD camera (Gatan, Japan) over a − 60° to + 60° tilt range (increment 1°) using automated tomography acquisition software (TEMography, 3-D Reconstruction Software, JEOL, Japan). Tilt series were aligned and computed into a tomogram using back-projection algorithms (TEMography).

### 3-D Segmentation, visualisation and sampling protocol

For 3-D modelling and visualisation, datasets were processed using IMOD a suite of image processing, modelling and display programs used for 3-D reconstruction and segmentation of tomographic data and EM serial sections^69^.

For AT datasets, cellular structures of interest were segmented by means of manual tracing of high-contrast lines using 3dmod, a graphical user interface application that is bundled with the IMOD software package^69^. Four cells from each patient were selected (16 cells in total), and each cell was divided into four equal planes (64 planes in total) along the *XZ* axis. In order to eliminate potential selection bias, all mitochondria intersecting one of the four *XY*-slicer images—tangential to the four *XZ* divisions for each cell—was segmented, rendered and classified either as a “normal” or “giant mitochondrion” on the basis of morphological features. For each HPC, the plasma membrane and associated nuclei were also modelled.

Of special note, in order to provide a more detailed visual example and morphometric illustration of an entire cell of interest, the total mitochondrial population, intracellular lipid droplets and HPC nuclei were also modelled (patient 4) .

For TET datasets, the inner and outer mitochondrial membranes were delineated by means of manual tracing, whilst intramitochondrial crystalline inclusions and enlarged matrix granules were segmented via semi-automated thresholding-based segmentation approaches. Visualisation of both AT and TET datasets of volume-rendered pseudocoloured structures of interest was performed within IMOD.

### Morphometry and statistical analysis

Quantitative measurements of a range of common morphometric parameters was performed on individual mitochondria using IMOD. Object measures including surface area and volume were obtained using the “imodinfo” script, whilst mitochondrial length and width measurements were obtained manually using the “measure tool”. For each patient, data are reported as means ± S.D. Two-tailed T-tests assuming unequal variance were used to evaluate differences between NM and GM, with the level of significance set at 0.05. Statistical analysis was performed using GraphPad Prism (version 7.02).

## Supplementary Information

Supplementary information 1.

Supplementary information 2.

Supplementary information 3.

Supplementary information 4.

Supplementary information 5.

Supplementary information 6.
