# Supplementary material for: Three-dimensional ultrastructure of giant mitochondria in human non-alcoholic fatty liver disease
Source: Sci Rep. 2021 Feb 8;11:3319. doi: 10.1038/s41598-021-82884-z (PMC7870882; doi:10.1038/s41598-021-82884-z)
Supplement: Supplementary file 1 — Supplementary information 1. [file 41598_2021_82884_MOESM1_ESM.pdf]

SUPPLEMENTARY INFORMATION

Three-dimensional ultrastructure of giant mitochondria in human non-alcoholic fatty liver disease

Gerald J. Shami, Delfine Cheng, Pauline Verhaegh, Ger Koek, Eddie Wisse & Filip Braet

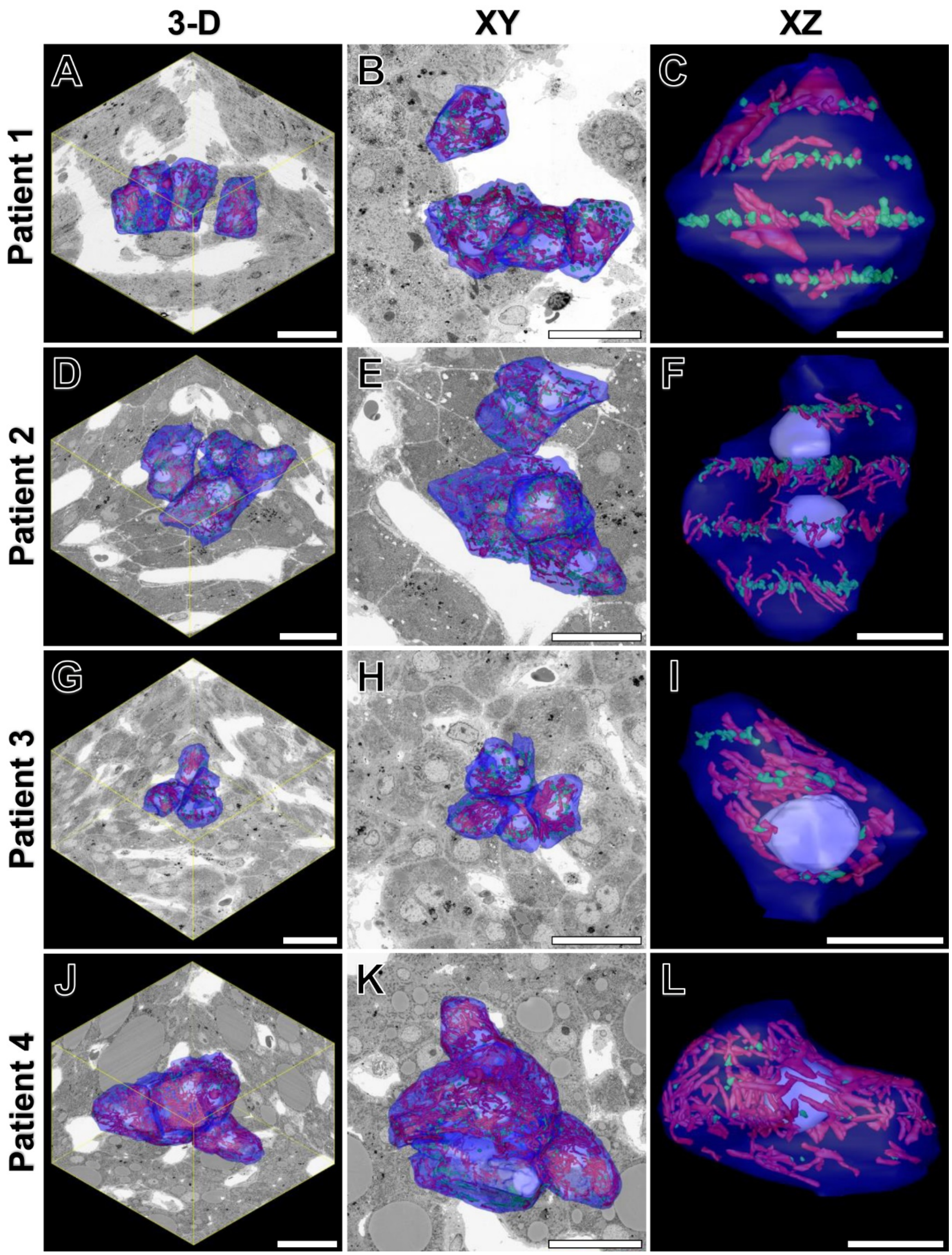

S1. (Left column) 3-D reconstruction of the hepatic microarchitecture of the four patients and corresponding model views of hepatic parenchymal cells (HPCs) (blue), normal mitochondria (green), giant mitochondria (red) and nuclei (white). (Middle column) 2-D slicer images overlayed with model views of the HPCs and associated subcellular structures corresponding to Column 1, 3-D. (Right column) Higher-magnification XZ model views of a single cell of interest selected from each dataset, revealing the sampling protocol employed. Each cell was divided into 4 equal planes along the XZ axis and all mitochondria intersecting one of the four XY-slicer images – tangential to the four XZ divisions – for each cell were modelled and classified as a normal- (green) or giant mitochondria (red). Scale bars: Column 1 & 2 = 25 µm; column 3 = 10 µm.

S2. Quantitative comparison of the cells analysed (*n* = 16), derived from each of the patients (*n* = 4), corresponding to the morphometric parameters summarised under Figure 2.

		**Patient 1**						
	**Cell**	**NM**			**GM**			
		**Mean**	**SD**	**SEM**	**Mean**	**SD**	**SEM**	***P* Value**
**Surface Area (μm^2)**	**1**	2.22	1.27	0.11	9.61	5.03	0.47	<0.0001
	**2**	2.32	1.24	0.11	8.45	4.74	0.52	<0.0001
	**3**	2.41	1.16	0.09	6.77	4.11	0.52	<0.0001
	**4**	2.27	1.17	0.07	7.91	4.49	0.38	<0.0001
**Volume (μm^3)**	**1**	0.24	0.16	0.01	1.29	0.88	0.08	<0.0001
	**2**	0.27	0.18	0.02	1.31	1.06	0.12	<0.0001
	**3**	0.31	0.20	0.02	1.10	0.88	0.11	<0.0001
	**4**	0.24	0.15	0.01	1.02	0.87	0.07	<0.0001
**SA:Volume**	**1**	10.97	3.02	0.26	8.18	1.61	0.15	<0.0001
	**2**	10.29	3.26	0.29	7.19	1.55	0.17	<0.0001
	**3**	9.06	2.57	0.20	6.54	1.02	0.13	<0.0001
	**4**	10.70	3.12	0.18	8.37	1.34	0.11	<0.0001
**Length (μm)**	**1**	1.22	0.58	0.05	4.07	1.57	0.15	<0.0001
	**2**	1.05	0.44	0.04	3.24	0.26	0.03	<0.0001
	**3**	1.02	0.40	0.03	2.52	0.20	0.03	<0.0001
	**4**	1.16	0.60	0.03	3.26	0.46	0.04	<0.0001
**Width (μm)**	**1**	0.52	0.12	0.01	0.73	0.23	0.02	<0.0001
	**2**	0.62	0.16	0.01	0.88	0.26	0.03	<0.0001
	**3**	0.70	0.17	0.01	0.95	0.20	0.03	<0.0001
	**4**	0.56	0.16	0.01	0.75	0.46	0.04	<0.0001
**Count**		**(N)**			**(N)**			
	**1**	137			114			
	**2**	125			84			
	**3**	158			63			
	**4**	299			139			
**Frequency (%)**	**1**	55			45			
	**2**	60			40			
	**3**	71			29			
	**4**	29			71			
		**Patient 2**						
	**Cell**	**NM**			**GM**			
		**Mean**	**SD**	**SEM**	**Mean**	**SD**	**SEM**	***P* Value**
**Surface Area (μm^2)**	**1**	2.37	1.16	0.06	8.47	4.23	0.28	<0.0001
	**2**	2.62	1.22	0.11	8.27	3.17	0.31	<0.0001
	**3**	2.66	1.28	0.09	8.02	3.51	0.42	<0.0001
	**4**	2.43	1.46	0.10	7.65	2.83	0.26	<0.0001

**Volume (µm^3)**	**1**	0.22	0.12	0.01	0.81	0.45	0.03	<0.0001
	**2**	0.26	0.12	0.01	0.77	0.32	0.03	<0.0001
	**3**	0.26	0.13	0.01	0.77	0.32	0.04	<0.0001
	**4**	0.22	0.14	0.01	0.72	0.29	0.03	<0.0001
**SA:Volume**	**1**	11.13	1.87	0.10	10.61	1.19	0.08	<0.0001
	**2**	10.58	1.75	0.16	10.83	1.26	0.12	^{ns}0.2099
	**3**	10.74	1.71	0.13	10.40	0.96	0.11	*0.0454
	**4**	11.54	1.75	0.12	10.86	1.45	0.13	<0.0001
**Length (µm)**	**1**	1.32	0.62	0.03	4.05	1.69	0.11	<0.0001
	**2**	1.19	0.54	0.05	4.11	1.38	0.13	<0.0001
	**3**	1.25	0.58	0.04	3.83	1.30	0.15	<0.0001
	**4**	1.29	0.79	0.05	4.04	1.34	0.12	<0.0001
**Width (µm)**	**1**	0.53	0.13	0.01	0.53	0.14	0.01	^{ns}0.5135
	**2**	0.51	0.11	0.01	0.52	0.12	0.01	^{ns}0.3826
	**3**	0.51	0.12	0.01	0.55	0.12	0.01	*0.0173
	**4**	0.50	0.10	0.01	0.55	0.11	0.01	<0.0001
**Count**		**(N)**			**(N)**			
	**1**	354			236			
	**2**	117			105			
	**3**	185			71			
	**4**	216			116			
**Frequency (%)**	**1**	60			40			
	**2**	53			47			
	**3**	73			27			
	**4**	48			52			
		**Patient 3**						
	**Cell**	**NM**			**GM**			
		**Mean**	**SD**	**SEM**	**Mean**	**SD**	**SEM**	***P* Value**
**Surface Area (µm^2)**	**1**	2.00	1.01	0.10	12.92	7.61	0.94	<0.0001
	**2**	2.54	1.37	0.16	9.50	7.26	1.09	<0.0001
	**3**	2.38	1.39	0.18	11.61	8.37	1.17	<0.0001
	**4**	2.39	1.44	0.12	8.75	6.00	0.63	<0.0001
**Volume (µm^3)**	**1**	0.21	0.13	0.01	1.91	1.61	0.20	<0.0001
	**2**	0.29	0.18	0.02	1.22	1.08	0.16	<0.0001
	**3**	0.25	0.18	0.02	1.51	1.19	0.17	<0.0001
	**4**	0.28	0.23	0.02	1.40	1.31	0.14	<0.0001
**SA:Volume**	**1**	10.74	2.69	0.27	7.84	1.63	0.20	<0.0001
	**2**	10.14	2.87	0.34	8.11	0.88	0.13	<0.0001
	**3**	10.75	2.70	0.34	8.01	0.99	0.14	<0.0001
	**4**	10.29	3.54	0.29	6.92	1.12	0.12	<0.0001
**Length (µm)**	**1**	0.97	0.40	0.04	4.31	1.95	0.24	<0.0001

	**2**	1.12	0.44	0.05	3.55	1.88	0.28	<0.0001
	**3**	1.17	0.53	0.07	4.11	2.03	0.28	<0.0001
	**4**	1.05	0.46	0.04	3.07	1.68	0.18	<0.0001
**Width (µm)**	**1**	0.59	0.13	0.01	0.79	0.28	0.04	<0.0001
	**2**	0.58	0.15	0.02	0.75	0.22	0.03	<0.0001
	**3**	0.54	0.13	0.02	0.73	0.21	0.03	<0.0001
	**4**	0.61	0.16	0.01	0.77	0.19	0.02	<0.0001
**Count**		**(N)**			**(N)**			
	**1**	98			65			
	**2**	72			44			
	**3**	62			51			
	**4**	144			91			
**Frequency (%)**	**1**	60			40			
	**2**	62			38			
	**3**	54			46			
	**4**	32			68			
		**Patient 4**						
	**Cell**	**NM**			**GM**			
		**Mean**	**SD**	**SEM**	**Mean**	**SD**	**SEM**	***P* Value**
**Surface Area (µm^2)**	**1**	1.29	1.33	0.10	10.85	7.20	0.35	<0.0001
	**2**	2.33	1.27	0.10	9.54	7.26	0.56	<0.0001
	**3**	1.99	1.18	0.14	10.27	6.64	0.53	<0.0001
	**4**	1.90	1.06	0.13	10.93	8.20	0.68	<0.0001
**Volume (µm^3)**	**1**	0.24	0.15	0.01	1.27	1.01	0.05	<0.0001
	**2**	0.21	0.12	0.01	0.97	0.86	0.07	<0.0001
	**3**	0.19	0.13	0.02	1.12	0.78	0.06	<0.0001
	**4**	0.19	0.13	0.02	1.45	1.41	0.12	<0.0001
**SA:Volume**	**1**	10.45	2.20	0.16	8.88	1.16	0.06	<0.0001
	**2**	12.18	2.65	0.21	10.20	1.49	0.11	<0.0001
	**3**	11.37	2.25	0.26	9.43	1.03	0.08	<0.0001
	**4**	11.76	2.90	0.35	8.31	1.47	0.12	<0.0001
**Length (µm)**	**1**	1.16	0.59	0.04	4.39	1.76	0.08	<0.0001
	**2**	1.31	0.67	0.05	4.12	1.66	0.13	<0.0001
	**3**	1.04	0.47	0.05	4.59	1.66	0.13	<0.0001
	**4**	1.15	0.54	0.06	4.07	1.40	0.12	<0.0001
**Width (µm)**	**1**	0.55	0.20	0.02	0.61	0.16	0.01	***0.0005
	**2**	0.47	0.08	0.01	0.53	0.14	0.01	<0.0001
	**3**	0.50	0.10	0.01	0.54	0.10	0.01	**0.0053
	**4**	0.51	0.09	0.01	0.69	0.24	0.02	<0.0001
**Count**		**(N)**			**(N)**			

	**1**	184	434	[redacted]
	**2**	159	170	
	**3**	75	159	
	**4**	70	145	
[redacted]				
**Frequency (%)**	**1**	29	71	
	**2**	48	52	
	**3**	32	68	
	**4**	33	67	

Legend: ns = P > 0.05; * = P ≤ 0.05; ** = P ≤ 0.01; *** = P ≤ 0.001. Unless otherwise indicated *P* = ≤ 0.0001.

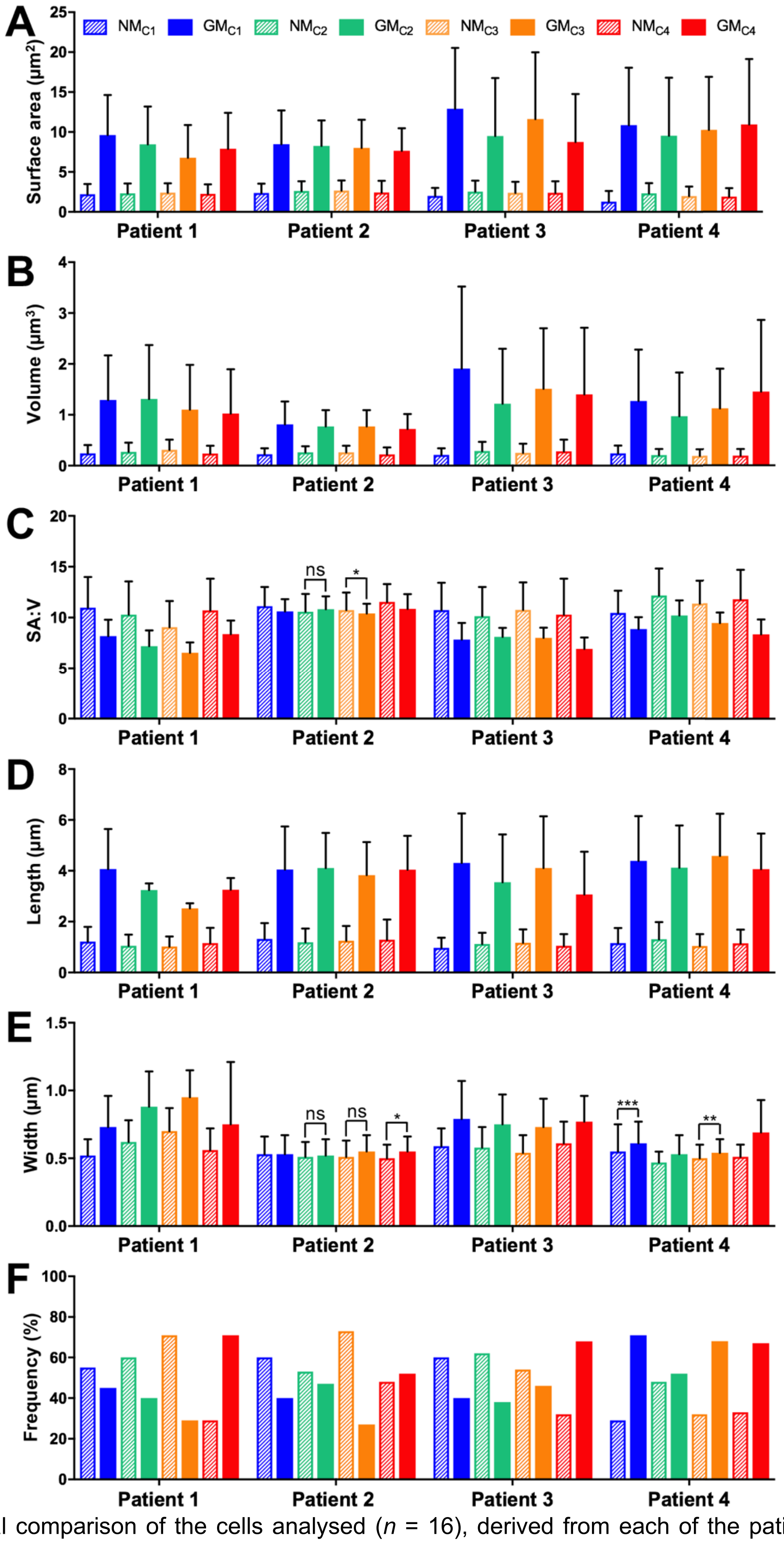

S3. Graphical comparison of the cells analysed (n = 16), derived from each of the patients (n = 4), corresponding to the morphometric parameters summarised under Figure 2 and S1. Legend: ns = $P > 0.05$; * = $P \leq 0.05$; ** = $P \leq 0.01$; *** = $P \leq 0.001$. Unless otherwise indicated $P = \leq 0.0001$.

S4. Tabular data corresponding to the patient summary graphs in Figure 2.

		**Patient 1**			**Patient 2**			**Patient 3**			**Patient 4**			**Combined Score**		
		**NM**	**GM**	***P***	**NM**	**GM**	***P***	**NM**	**GM**	***P***	**NM**	**GM**	***P***	**NM**	**GM**	***P***
**Surface Area (μm^2)**	**Mean**	2.30	8.33	<.0001	2.48	8.19	<.0001	2.32	10.54	<.0001	2.22	10.51	<.0001	2.35	9.51	<.0001
	**SD**	1.20	4.73		1.28	3.67		1.33	7.34		1.26	7.29		1.26	6.20	
	**SEM**	0.040	0.240		0.040	0.160		0.070	0.460		0.060	0.240		0.025	0.136	
**Volume (μm^3)**	**Mean**	0.26	1.17	<.0001	0.23	0.78	<.0001	0.26	1.52	<.0001	0.21	1.21	<.0001	0.24	1.13	<.0001
	**SD**	0.17	0.92		0.13	0.38		0.19	1.35		0.14	1.03		0.15	0.97	
	**SEM**	0.040	0.046		0.004	0.016		0.010	0.085		0.006	0.034		0.003	0.021	
**SA: Volume**	**Mean**	10.32	7.78	<.0001	11.08	10.68	<.0001	10.45	7.59	<.0001	11.34	9.14	<.0001	10.81	9.08	<.0001
	**SD**	3.09	1.58		1.82	1.24		3.07	1.31		2.57	1.4		2.63	1.78	
	**SEM**	0.115	0.079		0.062	0.054		0.158	0.083		0.117	0.046		0.053	0.039	
**Length (μm)**	**Mean**	1.12	3.37	<.0001	1.28	4.03	<.0001	1.06	3.69	<.0001	1.19	4.32	<.0001	1.18	3.99	<.0001
	**SD**	0.54	1.50		0.65	1.51		0.46	1.93		0.60	1.68		0.59	1.68	
	**SEM**	0.020	0.075		0.022	0.066		0.024	0.122		0.027	0.056		0.012	0.037	
**Width (μm)**	**Mean**	0.59	0.80	<.0001	0.51	0.54	***	0.59	0.76	<.0001	0.51	0.60	<.0001	0.55	0.64	<.0001
	**SD**	0.17	0.34		0.12	0.13	0.0004	0.15	0.23		0.51	0.17		0.15	0.24	
	**SEM**	0.006	0.017		0.004	0.005		0.008	0.014		0.007	0.006		0.003	0.005	
**Count (N)**		719	400		872	528		376	251		484	902		2451	2081	
**Frequency (%)**	**Mean**	64	36		62	38		60	40		35	65		54	46	
	**SD**															
	**SEM**															

Legend: *** = P ≤ 0.001. Unless otherwise indicated *P* = ≤ 0.0001.
