# Supplementary material for: Three-dimensional ultrastructure of giant mitochondria in human non-alcoholic fatty liver disease
Source: Sci Rep. 2021 Feb 8;11:3319. doi: 10.1038/s41598-021-82884-z (PMC7870882; doi:10.1038/s41598-021-82884-z)
Supplement: Supplementary file 2 — Supplementary information 2. [file 41598_2021_82884_MOESM2_ESM.docx]

**Supplementary Video 1.** Reconstructed 3-D volume consisting of 400 images corresponding to a cell of interest (blue) derived from patient 4. Giant mitochondria (red), normal mitochondria (green), nuclei (white) and lipid droplets (yellow) were rendered for 3-D visualisation.

**Supplementary Video 2.** 3-D reconstruction of an *elongated* giant mitochondrion, which were the most frequently occurring morphology. They were characterised by intracrystalline inclusions organised parallel to the longitudinal axis.

**Supplementary Video 3.** 3-D reconstruction of an *irregular* giant mitochondrion characterised by a branching or stellate morphology. Intracrystalline inclusions were observed in various orientations, running parallel to the various longitudinal axes of branching segments.

**Supplementary Video 4.** 3-D reconstruction of a *spheroidal* giant mitochondrion. These enormous structures displayed the most bizarre internal configuration, in which cristal membrane disorganisation was the most pronounced and intracrystalline inclusions were oriented in various directions through the mitochondrial matrix.
